# Elucidating Charge Generation in Green-Solvent Processed Organic Solar Cells

**DOI:** 10.3390/molecules26247439

**Published:** 2021-12-08

**Authors:** Safa Shoaee, Anna Laura Sanna, Giuseppe Sforazzini

**Affiliations:** 1 Disordered Semiconductor Optoelectronics, Institute of Physics and Astronomy, University of Potsdam, Karl-Liebknecht-Str. 24-25, 14476 Potsdam-Golm, Germany; 2 Dipartimento di Scienze Chimiche e Geologiche, Università degli Studi di Cagliari, Complesso Universitario di Monserrato, S.S. 554, Bivio per Sestu, I-09042 Monserrato, Italy; annal.sanna96@unica.it

**Keywords:** organic solar cells, green solvents, non-halogenated solvents, exaction diffusion, photoluminescence quenching

## Abstract

Organic solar cells have the potential to become the cheapest form of electricity. Rapid increase in the power conversion efficiency of organic solar cells (OSCs) has been achieved with the development of non-fullerene small-molecule acceptors. Next generation photovoltaics based upon environmentally benign “green solvent” processing of organic semiconductors promise a step-change in the adaptability and versatility of solar technologies and promote sustainable development. However, high-performing OSCs are still processed by halogenated (non-environmentally friendly) solvents, so hindering their large-scale manufacture. In this perspective, we discuss the recent progress in developing highly efficient OSCs processed from eco-compatible solvents, and highlight research challenges that should be addressed for the future development of high power conversion efficiencies devices.

## 1. Introduction

One of the greatest challenges to modern science is the search for new, clean and stable sources of energy which can provide for the growing requirements of an increasingly populated planet. Simultaneously, reducing damage to our natural environmental and halting man-made climate change is imperative. Indeed, one of the most pressing challenges of the 21st century is to reduce our net carbon emissions towards net zero to slow the effect of global warming. Thus, finding ways to generate electricity from sustainable sources and in a sustainable manner is pivotal to reaching this goal. To date, organic solar cells have shown an enormous potential as a viable candidate for green energy production. For instance bulk heterojunction (BHJ) organic photovoltaic (OPV), can be fabricated from solution at low-temperatures [1,2]. As a result, BHJ OPVs can be produced with a modest energy consumption [3,4] with respect to the high temperature approaches currently employed to process inorganic semiconductor materials (such as crystalline silicon). In addition, over the last decade, there has been substantial progress in the development of OPVs driven by advances in molecular design, material processing, and device engineering. Power conversion efficiencies (PCE) for the state of the art laboratory-scale devices exceed 18% for single junction cells [5] and are now approaching commercial viability. Thus, OPV is a promising technology with the potential to constitute a large portion of the future energy generation capability, but only if gains are made in performance, lifetime and eco-friendly fabrication.

The validated arrival of OPVs into the array of next-generation green-tech was promoted through tantalisingly attractive cost per power ($/W) values. These values would be achieved through leveraging the solubility of organic materials to diminish processing costs and fast production capacity through solution processes. To this date, approximately 30 years later, this plausibility has not been fully realized. However, given today’s public awareness on the health impact of product manufacturing, and the consciousness to prevent further damage to the environment and ecosystems, eco-friendly organic solar cells grant such attention [6]. Utilization of organic materials can guarantee an economically and environmentally sustainable life-cycle of the device if use of halogenated solvents in fabrication is removed. For instance, organic photovoltaics processed from green solvents demonstrate great potential for indoor photovoltaic applications, where toxicity should be eliminated. They can provide a self-suitable power source for Internet-of-Things (IoT) systems under indoor or low light intensity conditions [7,8]. Whilst crystalline silicon cells show low PCEs under indoor light, on the contrary, the highly tunable light-absorption properties of OPV photoactive materials combined with the environmentally friendly fabrication process, make them promising candidates for indoor applications. For this application, environmentally benign OPV address several inherent weaknesses in PV systems by enhancing design flexibility, environmental consideration, aesthetic demands and increasing efficiency under low-light conditions, leading to a smaller system footprint, particularly required by product designers and architects. Reporting in nature energy, Cui et al. demonstrated that 26% PCE can be obtained under a light emitting diode illumination [9]. Among a few studies [10,11,12]. Cutting et al. reported that the PCE of OPV devices can be increased up to 350% under LED light intensity relative to outdoor conditions [13]. This performance is significantly higher than that of competing Si-technologies or Perovskite-type cells under the same irradiance.

**Figure 1:**
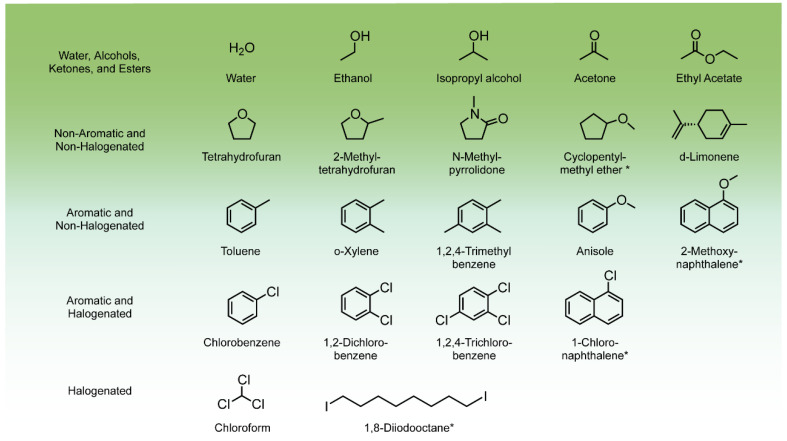
Representative examples of solvents used for BHJ solar cells organized in five general categories. Solvent hazard increases with the fading of green. Reviews about the toxicity and the environmental impact of the solvents have been recently published [16,20]. The mark ∗ indicates solvent additives.

Today’s cutting-edge PCE organic solar cells are commonly processed from halogenated and halogenated aromatic solvents, such as chloroform (CF) and chlorobenzene (CB), and might also include additive such as 1,8-diiodooctane (DIO) to achieve favorable film morphology of the light-harvesting active layer. Despite the worldwide spread of efforts to reach PCEs comparable to those obtained from halogenated-processed OSCs, to the best of our knowledge, eco-friendly organic solar cells are not there yet and the structure-function relationship is not explored or discussed. To date, several reviews have reported different chemistry aspects of green solvents [14,15,16] (Figure 1), and the impact of green solvents on the morphology of the OSC’s active layer [14,17,18,19]. Underpinning these structural considerations are the fundamentals of charge generation and collection. However, discussions on how these morphologies can be inherently linked with charge generation and recombination are currently lacking.

In this Perspective, we begin with a brief introduction to OSCs, discussing the principles for formation of free charges, and the requirement for a favorable morphology of the active layer. Then we will discuss the recent advances on the molecular design of organic semiconductors with enhanced solubility in eco-compatible solvents, and how the implemented molecular changes affect the film morphology and the efficiency of the device. Finally, we analyze the PCE in view of the photoluminescence quenching of OSCs processed with halogenated and non-halogenated solvent. The premise behind donor and acceptor materials and their role in exciton dissociation, and the boundary conditions that exciton diffusion lengths require for effective dissociation will also be explained. Finally, we highlight the critical aspects that should be solved for the future development of OSCs processed with sustainable solvents.

## 2. Introduction to OSCs

Organic solar cells with a bulk heterojunction architecture consist of an active layer with phase-segregated domains of the electron donor and the acceptor components [21]. Such BHJ solar cells are commonly prepared by combining conjugated donor (D) polymers with electron-accepting (A) molecules. This leads to an interpenetrating network with a large D-A contact area, where the absorbing site is within a few nanometers of the donor-acceptor interface. When light is absorbed by the donor (or the acceptor) an ‘exciton’ (LE) is generated, which can be regarded as an electron-hole pair bound together by electrostatic interactions. The first step for efficient energy transduction requires dissociation of the neutral excited state, localized on a donor or acceptor component, into a charge-transfer exciton that is localized on adjacent donor and acceptor components. and subsequently into long-lived free charges with a high quantum yield and minimal loss of free energy. Thus, a potential concern is that the electron and hole must overcome their mutual Coulomb attraction, *V*.

(1) $$V=\frac{e^{2}}{4\pi r\varepsilon_{r}\varepsilon_{0}}$$

where *e* is the charge of an electron, $\varepsilon_{r}$ is the dielectric constant of the surrounding medium, $\varepsilon_{0}$ is the permittivity of vacuum, and r is the electron-hole separation distance. For organic materials, overcoming this Coulomb attraction is demanding due to both small dielectric constant ($\varepsilon_{r}$ ≈ 2–4) and the localized nature of the electronic states involved. As such, achieving efficient charge photogeneration is a key challenge for solar energy conversion technologies based upon molecular materials.

**Figure 2:**
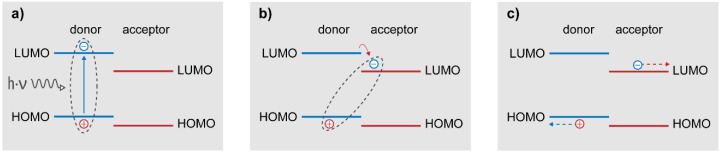
Description of the fundamental processes in OPV devices: (**a**) local exciton generation in the donor, (**b**) charge transfer state formation, and (**c**) free carrier formation and charge collection.

The events that occur in organic solar cells upon light illumination can be summarized as (Figure 2):

- Local exciton generation;
- Exciton diffusion to the donor/acceptor interface within its lifetime;
- Exciton dissociation at the D/A interface and charge transfer (CT) state formation;
- Separation of the CT state into free carriers;
- Charge carrier collection in the selective electrodes.

Bulk heterojunctions utilise pre-deposition solution-based mixing, with the objective of forming a randomly configured arrangement with length scales of the exciton diffusion length-order. The convoluted networks do not guarantee a percolating pathway to the collection points. Whilst largely unpredictable and susceptible to reproduction difficulties, this approach has become a field-wide standard and BHJs have consistently produced leading efficiency devices. Until recently only a small number of acceptors have proven capable of delivering high power conversion efficiencies. In particular until recently (2015) the fullerenes dominated the landscape. However since, non-fullerene acceptors (NFA) have delivered advances in cell efficiencies [22]. One of the most efficient systems, discovered in 2019, relies on the combination of the donor polymer PM6 with the small molecule NFA Y6 [23].

## 3. Morphology of the Photoactive Layer

**Figure 3:**
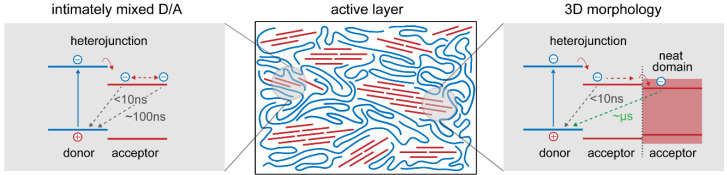
Illustration of the functional model for charge generation and recombination.

The key to making efficient OSCs is to ensure that the two materials are intermixed at a length scale less than the exciton diffusion length (typically 5–10 nm) so that every LE formed can reach an interface to undergo charge transfer. At the same time, the morphology has to enable charge-carrier transport in the two different phases to minimize recombination (Figure 3). This *morphology* is determined by the *processing solvent*, concentration, miscibility and crystallinity of D and A, as well as other parameters. Depending on the degree of miscibility and crystallinity between donor and acceptor, 2D or 3D microstructures (the pure D and or A phases, and the D/A amorphous intermixed phase) describe the morphology of the active layer [24]. A certain amount of mixed amorphous phases is crucial for efficient charge generation, while the phase-separated morphology is known to be critical for charges to be both stabilized and extracted. The combination of both factors will determine the final photocurrent of the device. When the donor–acceptor miscibility is too high (hyper-miscible) it can lead to performance deteriorations due to insufficient phase separation to allow stabilization of carriers (to avoid recombination). On the other hand, a miscibility that is too low (hypo-miscible) leads to limitation in charge generation [25]. In this regard, the choice of solvent, which influences the solubility as well as miscibility, can play a significant role on the morphology. Since organic semiconductors are typically made from extended aromatic sub-units, most high performing devices are processed from halogenated solvents (e.g., chloroform, o-dichlorobenzene) which provide good solubility and thereby miscibility of D and A.

There have been a lot of successful attempt in device efficiencies and numerous studies of photoinduced charge separation of fullerene and non-fullerene acceptor based photoactive layers processed from halogenated solvents [26,27,28,29,30]. Extensive studies of these systems have led to a detailed understanding of their structure/function relationship in terms of nonadiabatic electron transfer theory [31]. It has been shown, for example, that charge photogeneration in most systems is dependent upon sufficient domain size and miscibility, whilst the use of energetic cascade (due to aggregation) is required to increase their spatial separation and avoid undesired recombination [24].

The use of halogenated solvents allows, in principle, through good miscibility, the formation of percolation pathways to achieve the electrical ‘wiring’ of charge photogeneration at the donor/acceptor interface to external device electrodes. Active layers processed from green solvents on the other hand, typically do not exhibit high miscibility between the donor and the acceptor that is present in aromatic halogenated solvents. The mechanism by which active layers processed from green solvents can overcome the Coulomb attraction of the photogenerated electron-hole pair, and in particular achieve this with high quantum efficiency, is central to the development of green organic solar cells.

**Figure 4:**
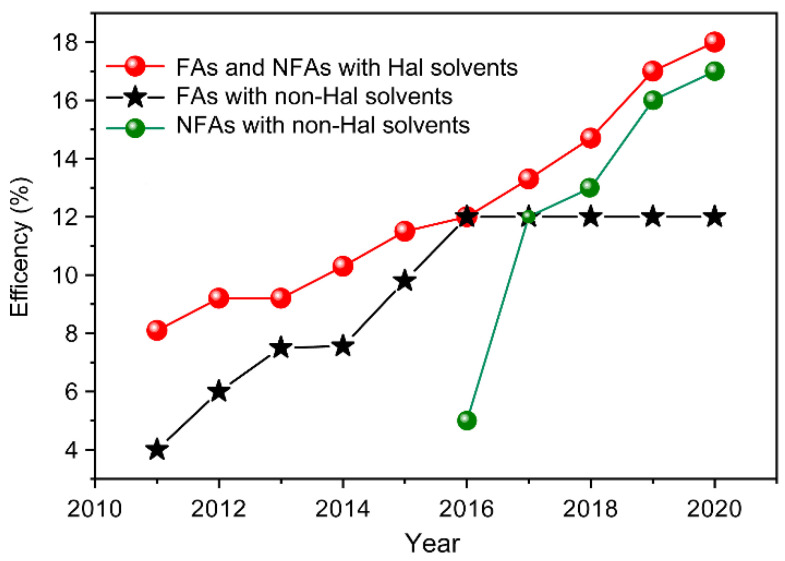
Trends in performance between halogenated and non-halogenated processed organic solar cells.

To date, only a few publications have attempted to use green solvents (Figure 1), and until recently most non-halogenated solvent processed devices deliver inferior performance to the halogenated solvent processed ones (Figure 4) [14,32,33,34,35]. This is due to either solubility limitations and/or other morphological aspects, such as interactions of the solvents and components, as well as the rate of solvent evaporation. These have been addressed extensively in recent reviews [19]. In this perspective we focus on the implication of the morphology on charge generation.

One limitation with respect to OSCs efficiency processed from green solvents stems from the short exciton diffusion length. The problem with morphology due to miscibility is typically expected to reflect on inefficient exciton dissociation. Efficient device performance relies upon the photogenerated exciton moving to a donor/acceptor interface so that exciton dissociation can occur [36,37]. Due to their electrical neutrality, the motion of excitons is not affected by electric fields, and thus they diffuse through the blend randomly. In this regard, an important parameter of excitons is the diffusion length: the distance an exciton can migrate before relaxing back to the ground state [31,38]. Dissociation of the exciton into charges must therefore occur within this distance. Clearly, this will limit the extent of phase segregation possible in a bulk heterojunction blend morphology for efficient device performance. In general, phase segregation on the order of the exciton diffusion length is desired. Measurements of exciton diffusion length have yielded values of 5-14 nm [37,39,40] for most conjugated polymers and [6,6]-phenyl-C61-butyric acid methyl ester (PCBM), but some recent NFAs exhibit values up to 50 nm long [41].

## 4. Organic Semiconductors Design towards Green Solvent Processing OSCs

**Figure 5:**
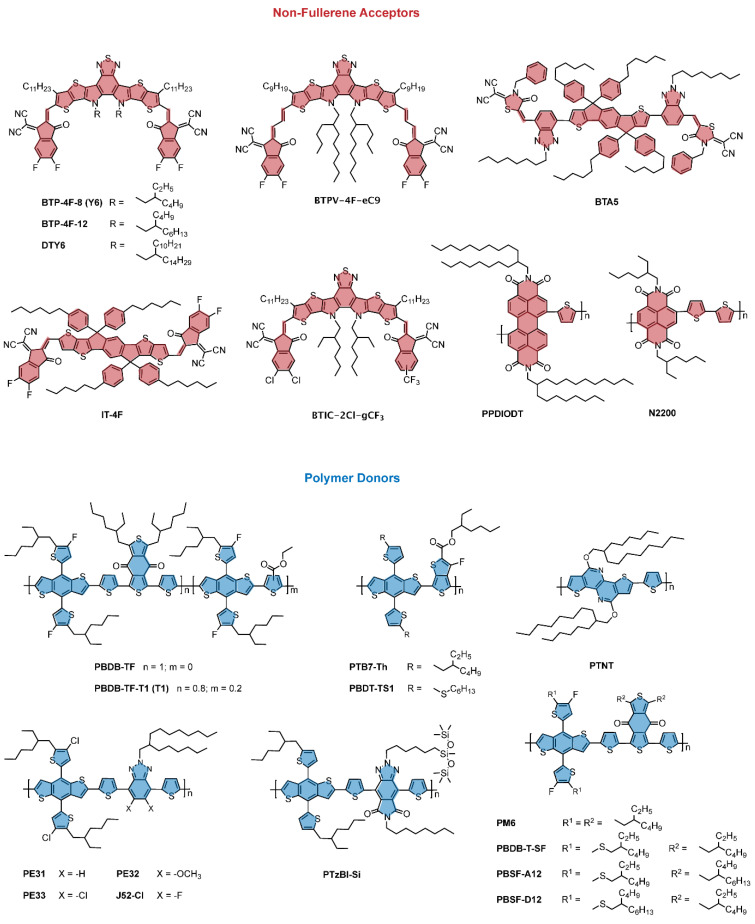
Selected representative examples of NFAs and polymer donors used in BHJ solar cells processed from non-halogenated solvents.

To prepare high performance OSCs using green solvents it is necessary to achieve a film morphology that is favorable for charge separation and transport. This requires the coexistence of both intimately mixed D and A regions as well as phase segregated domains [22,42]. Thus, it is imperative to finely tune the design of semiconductors so as to allow for a good miscibility, and at the same time to govern the packing of the molecules in the solid state. However, finding the balance among molecule solubility, material crystallization and phase separation is not trivial. As solubilizing chains are pivotal for the processing of the materials, the intermolecular arrangement of the semiconductors is strongly influenced by them when processed into films. As a result, the electronic properties of the materials are affected by the choice of the side chains. Moreover, to achieve high PCEs the semiconductors constituting the D/A active layer should have complementary absorption to harvest most of the solar light, whilst at the same time exhibiting favorable molecular energy levels. Fullerene-based electron acceptors commonly suffer from poor absorption in the visible and NIR portion of the solar spectrum, tunability of its energy level, and solubility in polar media. Attempts to improve these chemical and physical properties by dedicated molecular engineering usually result in materials that underperform with respect to the classic PCBM. On the other hand, NFA have raised attention due to their molecular design tailoring, which allows for improvement of both processability and optoelectronic properties. Nonfullerene acceptors are commonly designed on an acceptor–donor–acceptor (A–D–A) type structure [23]. NFA backbones consist of electron-donating fused-polycyclic systems with terminal electron-accepting units, and solubilizing chains on sides (Figure 5). Such a molecular architecture is responsible for the rapid progress on the tuning of both molecular electronic properties and processability in non-halogenated solvents. In this context, two of the most commonly adopted approaches rely on the introduction of dedicated side chains and the enhancement of the dihedral angle between the aromatic subunits. Following the increasing interest in NFAs, considerable research effort has been also devoted to design and synthesis of polymer donors with an improved solubility in green solvent. Randomizing of copolymer backbone by incorporating a third monomeric unit to design terpolymer, as well as varying the regioregularity or tuning the steric interactions of lateral substituents have been proven to be among the most successful strategies (Figure 5).

An NFA electron acceptor processable from green solvent was synthesized by *Hong* et al. by replacing the 2-ethylhexyl side chains of Y6 with longer 2-butyloctyl groups, so as to design BTP-4F-12. Such a change allowed for a good solubility in non-halogenated solvents such as o-xylene (o-XY), 1,2,4-trimethylbenzene (TMB), and tetrahydrofuran (THF), as well as for an improved in-plane intermolecular stacking which facilitates charge transport. The same group prepared a modified version of PBDB-TF by introducing an ester-substituted thiophene as a third polymer repeating unit. The resulting polymer, named T1, exhibits an improved solubility in non-chlorinated solvents due to the twisting of the backbone induced by steric interactions of its lateral substituents. OSCs fabricated from blends of BTP-4F-12:T1 processed in THF exhibit PCE of 16.1% which is comparable to the values obtained for CF-processed devices [34]. Recently, Jia et al. conducted a chemical modification for Y6 by inserting a second ethylene double bond π-bridge between the central fuse ring and the terminal indane derivative [43]. The resulting structure BTPV-4F exhibits an absorption that covers a larger portion of the solar spectrum than the original Y6, so as to improve its absorption also in the NIR. To render BTPV-4F suitable for non-halogenated solvents, Qin et al. replaced the 2-ethylhexyl group with 2-butyloctyl side chains, to design BTPV-4F-eC9 [44]. OSCs made from THF-processed binary blends of the latter with PTB7-Th exhibit PCEs of 12.77% that are higher than those obtained using chloroform and 1-chloronaphthalene additive. Chen et al., designed a dissymmetric version of Y6 by replacing the two fluorine atoms with two of chlorine in one terminal indane derivative, and by introducing a trifluoromethyl group in the other side of the molecule, instead of the two fluorine atoms. The corresponding compound, named BTIC-2Cl-γCF_3_, exhibits an enhanced solubility in toluene and an ability to form a well-organized packing network in the solid state. Binary blend of BTIC-2Cl-γCF3 and PBDB-TF processed from toluene afford devices with power conversion efficiency as high as 16.31% [45].

In order to increase the solubility of PBDB-T-SF, an analogue of electron donating polymer PM6, Wang et al. extended the side chains on the donor and acceptor repeating units from 2-ethylhexyl to 2-butyloctyl in order to design PBSF-D12 and PBSF-A12, respectively. As a result, the new wide band-gap polymers have a good solubility in toluene. Films PBSF-D12 exhibit red-shifted absorption spectra due to a high degree of molecular organization which is beneficial for the charge mobility. Toluene processed the OSCs based on PBSF-D12:IT-4F exhibited a good photovoltaic performance with a PCE of 13.4% [46]. Dai et al. conducted modifications for J52-C by replacing the fluorine atoms of the benzotriazole unit with hydrogen atoms, methoxy groups, chlorine atoms. The resulting compounds PE31, PE32, PE33 were tested in THF-processed OSCs made from binary blends with BTA5. Devices resulting from the unsubstituted polymer PE31 achieved the highest PCE of 10.08%. Such a result is ascribed to a better packing of the compounds leading to a good level of intermolecular organization. In contrast, due to the weak crystallinity of the methoxy-substituted polymer PE32, the resulting device shows the lowest PCE of 7.40% [47]. All polymer solar cells were prepared by Sunsun et al. using a perylenediimide (PDI)-bithiophene-based polymer acceptor PPDIODT, and PBDT-TS1 as polymer donor. The former polymeric NFA is designed to have large dihedral angles between its thiophene and PDIs subunit, and to hold a 2-octyldodecyl chain on the PDI motif. As a result, PPDIODT exhibits a good solubility in various green solvents, such as toluene and anisole. OSCs fabricated from binary blend of PPDIODT:PBDT-TS1 processed from anisole have recorded a very good PCE of 6.58% [48]. Polymer donors with enhanced solubility in non-halogenated solvents were also prepared using siloxane-functionalized side-chains. Fan et al. designed and synthesized PTzBI-Si, an electron-donating polymer containing amide-functionalized benzotriazole inclusive of the siloxane-based solubilizing group. The resulting polymer has good solubility in green solvent such as tetrahydrofuran (THF), 2-methyl-tetrafuran (2-MeTHF), and cyclopentyl methyl ether (CPME). Moreover, at the solid state, PTzBI-Si assumes preferentially face-on orientation and forms optimal morphology so as to facilitate carrier mobility in devices. All-PSCs were fabricated using binary blends of PTzBI-Si and the polymer acceptor N2200, processed from THF, 2-MeTHf and CPME, delivering the a slightly higher PCE of 11.0% with the latest solvent [33].

Besides the aforementioned examples, another approach to fabricate OSC from environmentally benign solvents is to anchor the organic semiconductors onto the surface of nanoparticles [49,50]. The later enables pre-aggregation of the donor and the acceptor domains and achieves phase separation, forming a beneficial BHJ morphology [51]. However this technology is underdeveloped and only a few cases of donor and acceptor combinations that can use this method have been reported so far [52].

The large number of variable involved in the fabrication of a solar cell, such as the different chemical structure of the donor and acceptor, the presence of various solubilizing side groups, the use of different processing techniques, as well as the different nature of media used to cast the active layer, render it difficult to quickly assess the structure-property-performance relationships of the devices. Thus, to deepen the origin of the better performances of halogenated-processed OSCs over their eco-compatible counterpart it is essential to analyze charge generation.

## 5. Photoluminescence Assay of Exciton Dissociation

The primary experimental technique employed to assay the efficiency of exciton dissociation in excitonic D/A blend films is photoluminescence (PL). This is a straightforward technique that monitors the yield of emission of excitons in the blend compared to the pristine material. Quenching of the radiative emission in the blend insinuates exciton dissociation.

Whilst in halogenated processed active layers domain size smaller than the order of exciton diffusion length has been achieved, for non-halogenated and green solvent processed active layers, with hypo-miscible morphology, domains of D and A can be larger than the exciton diffusion length. Thus, the exciton which is photogenerated in one domain, cannot completely diffuse to an interface for dissociation, before relaxing back to the ground state. In this case, only the fraction of excitons generated within the exciton diffusion length will dissociate to form an interfacial CT state and potentially contribute to the photocurrent.

**Figure 6:**
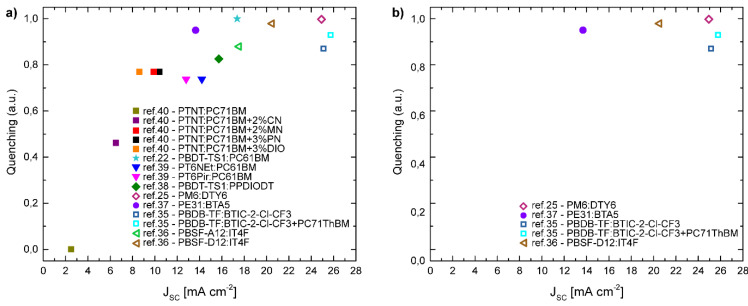
(**a**) Photoluminescence quenching as a function of photocurrent at short circuit, J_SC_, for a number of fullerene and non-fullerene acceptor based solar cells processed from non-halogenated solvents [32,35,45,46,48,53,54,55]. Halogenated and non-halogenated additives: 1-Methoxynaphthalene (MN), 1-Phenylnaphthalene (PN), 1-Cloronaphthalene (CN), 1,8-Diiodooctane (DIO). (**b**) A selection of data from panel (a) which exhibit high quenching.

This is exemplified in the work by Lanzi et al. who designed and synthetized water-soluble polythiophenes by introducing hexyl side chains with terminal aminium group (PT6NEt^+^), and with pyridine unit (PT6Pir), to be blended together with PCBM. From photoluminescence studies it was shown that only about 70% of the excitons reach a D/A interface to dissociate, thus limiting charge generation [53]. This cannot, in general, be attributed to unfavorable energetics. Rather, this efficiency loss appears to derive from the tendency of the blend to form D or A aggregates (large domains) on the length scale or larger than their exciton diffusion lengths. Such a scenario is consistent with what has also been observed in D:A blends with high crystalline domains. In this regard it is anticipated that with the new generation of NFAs which have exceeded previous numbers, such statements and questions of the processes limiting device efficiency are susceptible to revision. Indeed, there are a handful of papers using green solvents, which have investigated the efficiency of exciton diffusion to the interface and thereby its dissociation. Li et al., reported an all polymer solar cell using perylene diimides PPDIODT blended with PBDT-TS1, processed from anisole. In these samples, strong PL quenching of the blend compared to the neat was observed; indicating domain size compatible with exciton diffusion length [48]. Similar observation was reported for the study of benzotriazole (BTA)-based p-type polymers (PE31, PE32, PE33, and J52-Cl) when blended with a BTA-based small molecule BTA5 using THF [47]. It was shown that PE31:BTA5 exhibited the highest PCE of 10.08%, which correlated with the highest PL quenching, whilst the other blends had reduced PL quenching; insinuating less optimum morphology of domain size. One of the most attractive systems reported yet is the PM6:DTY6 blend which shows close to 100% PL quenching and an efficiency of 16% when processed from xylene. Figure 6a plots photocurrent at short circuit against photoluminescence quenching. Correlations between two parameters suggest that the miscibility and molecular organization between the polymer and the acceptor are a key consideration for optimization of photocurrent generation and indicative of exciton diffusion limitations.

Charge separation in donor/acceptor blend systems can be most simply described as electron transfer from excitons to generate free charges. In this simple picture, the efficiency of exciton quenching at the D/A interface should correlate directly with the yield of photogenerated charges. Whilst the general trend in Figure 6a indicates that the photocurrent is limited by exciton quenching (due to too large domains), however upon a closer examination of the systems and categorization of the different class of acceptors, in Figure 6b we observe photocurrent to be independent of the exciton quenching values in the NFA based systems.

Indeed the fused-ring NFA materials benefit from higher film crystallinity (due to better π–π molecular packing) and lower energetic disorder [56]. The higher crystallinity has synergy in enhancing exciton and of free carrier diffusion length (to reach an interface and thereby dissociate) as well as suppressed recombination of free carriers due to crystalline pure phases [57,58]. The lower energetic disorder can also aid exciton dissociation. Excitons are electrically neutral and diffuse through the blend randomly. This diffusion is typically described as a Forster-type incoherent energy transfer process, which can be either intramolecular or intermolecular and usually acts to lower the energy of the exciton. This downhill energy transfer can result in trapping of the exciton in the tail of the inhomogeneously broadened density of states, where the trap sites are often associated with defects and aggregates. At this point, any further exciton migration will rely on thermal fluctuations. In the new class of NFAs, these defects are typically much smaller; 60 meV compared to 120 meV in the PCBM systems [59], thus allowing for more excitons to be able to benefit from thermal fluctuations in order to achieve exciton dissociation at an interface.

Figure 6b implies that, for this materials series, exciton quenching is not the limiting factor for charge photogeneration: the efficiency of the CT-state dissociation into free charges is instead. Thus in addition to the exciton dissociation, the degree of miscibility between the donor and the acceptor still plays a role in CT dissociation and recombination of the free carriers. It is well established that a 3D morphology consisting of a mixed phase as well as pure phases is required to initially generate carriers but to also spatially stabilise the free carriers from one another [24,60]. Thus, in the cases where green solvent prevents coexistence of intimately mixed as well as aggregated phases, the CT state dissociation efficiency is either inefficient or requires a new mechanism for generation of free carriers. Whilst the field is being driven forward in terms of numbers, there is however very limited fundamental studies, elucidating the loss and working mechanisms in green solvent processed organic solar cells. Whilst the initial reports of non-halogenated solvent processed solar cells is encouraging; however, further and general enhancement warrants a deeper understanding of the limiting steps.

## 6. Concluding Remarks

The proceeding sections showcased the constant development of novel materials rapidly pushing forward the PCEs of OSCs. However, the efficiencies of devices processed via eco-compatible solvents have not yet coherently reached the performance of the OPVs processed by halogenated solvents. To reach the commercialization of OSCs, eco-compatible solvents have to replace toxic organic solvents. Herein, we reviewed current state of the art efficiencies of organic solar cells when processed from non-halogenated solvents. In addition, we discussed the chemical structure and some of the performance-limiting steps of OSCs processed from eco-compatible solvents. A recurrent theme in the current literature is the role of morphology for device performance. Many studies and other reviews have addressed the influence of blend nanomorphology on the device performance. However, studies relating morphology to charge photogeneration remain rather indirect and very few. Nevertheless, from the few studies, experimental evidence is slowly accumulating that exciton dissociation is not the limiting photocurrent as exemplified by PM6:DY6 [35]. However, in order to get an accurate and reliable predictive model to relate materials’ structures to photovoltaic device performance understanding the limiting steps in charge generation and recombination is deemed necessary.

## Data Availability

Not applicable.
